# Cranial cerebrospinal fluid leak and intracranial hypotension syndrome – a case report

**DOI:** 10.25122/jml-2021-0090

**Published:** 2021

**Authors:** Razvan Alexandru Radu, Elena Oana Terecoasa, Andreea Nicoleta Marinescu, Iulian Enache, Cristina Tiu

**Affiliations:** 1. Department of Neurology, University Emergency Hospital Bucharest, Bucharest, Romania; 2. Department of Neurology, Carol Davila University of Medicine and Pharmacy, Bucharest, Romania; 3. Department of Radiology and Medical Imaging, University Emergency Hospital Bucharest, Bucharest, Romania; 4. Department of Radiology and Medical Imaging, Carol Davila University of Medicine and Pharmacy, Bucharest, Romania

**Keywords:** intracranial hypotension, cranial dural leak, orthostatic headache, temporal bone fracture, CSF – cerebrospinal fluid, CT – computed tomography, ENT – otorhinolaryngology, MRI – magnetic resonance imaging, SIH – spontaneous intracranial hypotension

## Abstract

Spontaneous intracranial hypotension is a rare clinical entity caused in most cases by a cerebrospinal fluid leak occurring at the level of the spinal cord. Cranial dural leaks have been previously reported as a cause of orthostatic headaches but, as opposed to spinal dural leaks, were not associated with other findings characteristic of spontaneous intracranial hypotension. We present the case of a male admitted for severe orthostatic headache. The patient had a history of intermittent postural headaches, dizziness, and symptoms consistent with post-nasal drip, which appeared several years after head trauma. Brain imaging showed signs consistent with intracranial hypotension: bilateral hygromas, subarachnoid hemorrhage, superficial siderosis, diffuse contrast enhancement of the pachymeninges, and superior sagittal sinus engorgement. No spinal leak could be identified by magnetic resonance imaging, and the patient had a rapid remission of symptoms with conservative management. Further work-up identified an old temporal bone fracture which created a route of egress between the posterior fossa and the mastoid cells. Otorhinolaryngology examination showed pulsatile bloody discharge and liquorrhea at the level of the left pharyngeal opening of the Eustachian tube. The orthostatic character of the headache, as well as the brain imaging findings, were consistent with intracranial hypotension syndrome caused by a cranial dural leak. Clinical signs and imaging findings consistent with the diagnosis of apparently “spontaneous” intracranial hypotension should prompt the search for a cranial dural leak if a spinal leak is not identified.

## Introduction

Orthostatic headache is the hallmark finding of spontaneous intracranial hypotension (SIH). The headache is usually severe, bilateral, appears a few moments after standing up, and improves by lying down. It may be associated with nausea, vomiting, neck pain, vertigo, tinnitus, and other signs and symptoms related to dysfunction of cranial nerves [1].

Leakage of cerebrospinal fluid (CSF) through a defect in the cervicothoracic thecal sac is the most frequently identified cause of SIH [1]. In 44–55% of cases, a spinal leak may not be identified by imaging techniques, but empiric treatment with lumbar epidural blood-patch or microsurgical exploration usually settles the case [1, 2]. Up to now, there is no clear evidence that cranial dural CSF leaks cause SIH despite several reports describing orthostatic headache in patients with cranial CSF leaks [1, 3].

## Case Report

We report the case of a 76-year-old Caucasian man who presented to our hospital for acute onset epistaxis associated with headache and dizziness. He had a history of arterial hypertension, left bundle branch block, dyslipidemia and heart failure, for which he was taking antihypertensive and antiplatelet drugs. He also reported a traumatic head injury in the context of a road traffic accident seventeen years before, after which he had otorhinolaryngology (ENT) surgery twice: the first time for traumatic fracture of the nasal pyramid and second time to correct a nasal septal defect.

**Figure 1.**
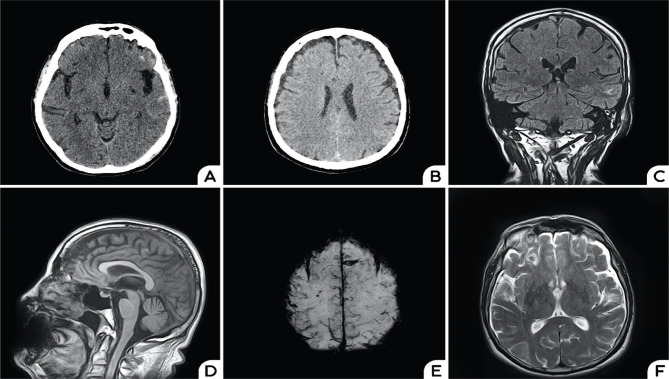
Axial native CT scan showing subarachnoid hemorrhage located in the frontal lobes bilaterally and in the left temporal lobe (A); Follow-up CT scan performed 48h later showing bilateral hygromas (B); Coronal FLAIR showing superior sagittal sinus engorgement, dilated cortical veins, hemorrhage in the left temporal lobe (C); Sagittal T1-weighted imaging showing sunken splenius (D); Axial SWI showing superficial siderosis (E); Axial T2-weighted imaging showing hygromas and hemorrhage in the right frontal lobe (F).

Brain computed tomography (CT) showed small, subtle areas of subarachnoid hemorrhage located in both frontal and left temporal lobes and bilateral 5mm hygromas (Figure 1). A subsequent CT angiography ruled out an aneurysm, and the patient was admitted to the Department of Neurology.

General clinical examination was unremarkable. Blood pressure was 155/80 mmHg and his heart rate was regular at 80 beats per minute (bpm). Neurological examination showed a left-sided peripheral facial palsy (sequela from his car accident) without any other pathological findings. Headache and dizziness occurred within minutes of adopting an orthostatic position and significantly improved when lying supine. His epistaxis resolved within hours from the onset and the otorhinolaryngology exam showed evidence of hemotympanum, considered to be a consequence of epistaxis. A thorough anamnesis revealed that he had a few episodes of isolated headache attacks sometimes associated with dizziness over the previous years, all presenting a strictly orthostatic character. These episodes were sometimes associated with symptomatic episodes consistent with post-nasal drip.

The brain magnetic resonance imaging (MRI) was consistent with the diagnosis of SIH, showing bilateral subdural hygromas, diffuse contrast enhancement of the pachymeninges, superior sagittal sinus engorgement, and small areas of cortical and subarachnoid hemorrhage in the left temporal and right frontal regions. Susceptibility weighted imaging showed evidence of superficial siderosis at the level of the frontal lobes bilaterally, which could be due to past episodes of subarachnoid hemorrhage or a consequence of his past head trauma (Figure 1).

**Figure 2.**
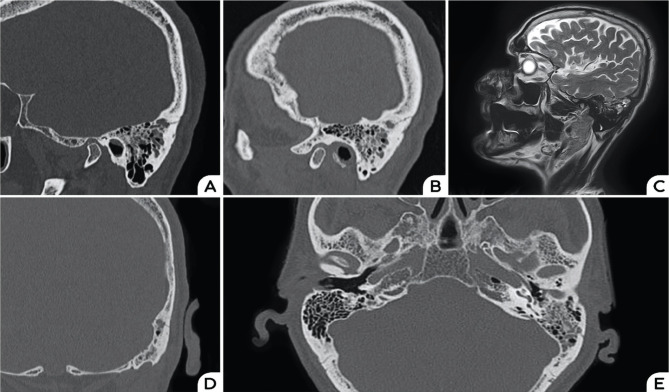
Sagittal hr-CT reconstruction of the temporal bone showing bone fracture involving the mastoid cells (A) and (B); Sagittal T2- weighted MRI showing hyperintense signal in the left mastoid cells (C); hr-CT scan reconstruction in coronal and axial planes showing the fracture involving an antral cell (D) and partial opacification of the left mastoid cells (E).

The patient was confined to bed rest and received an increased hydration regime, generous caffeine intake, and low dose dexamethasone (8 mg/day). Two spinal cord MRI scans aiming to identify a potential CSF leak were performed on separate days, but no signs of dural leakage were identified. His headache and dizziness progressively improved, but two days after admission, he complained of left ear fullness. A high-resolution CT scan of the temporal bone showed an oblique fracture involving the left temporal bone and communicating with the posterior superior marginal mastoid cell. Liquid density was visualized in the left mastoid cells, and therefore, a new brain MRI with fine T2-weighted sequences was performed, confirming this finding (Figure 2).

Repeated and more elaborate ENT examination showed left hemotympanum with intermittent pulsatile discharge from the pharyngeal opening of the Eustachian tube, and nasal endoscopy revealed oto-liquorrhea. However, repeated nasal endoscopy performed two days later, intending to obtain a sample for b2-transferrin testing, was unsuccessful because the discharge had stopped. Besides ear fullness, the patient remained symptom-free and was discharged home. He was further referred for ENT and neurosurgical follow–up in an experienced center for further assessment of the opportunity of surgical exploration aimed to clearly localize and seal the cranial dural defect and the temporal bone fracture. He did not report any other symptoms in the first month after discharge and refused surgery proposed by a joint neurosurgical-otorhinolaryngology team.

## Discussion

This is a rare case of intracranial hypotension syndrome associated with a cranial dural leak. We hypothesize that our patient developed intermittent slow-flow CSF leak, which led to atypical symptoms and an episodic occurrence of orthostatic headaches after the road accident. Episodic and intermittent orthostatic headaches have been described in patients with slow-flow CSF leaks before [1, 4]. Moreover, his brain MRI showed evidence of superficial siderosis and minimal subarachnoid hemorrhage. Superficial siderosis is uncommon in acute cases of SIH, but it can be the consequence of chronic stretching and repeated bleeding from the superficial bridging veins during acute episodes of CSF volume depletion [5, 6].

Ear fullness, symptoms of post-nasal drip (due to liquorrhea) as well as headaches have been reported in patients with temporal bone fractures, and these symptoms might sometimes be present for years before diagnosis [7]. It is estimated that 15% of temporal bone fractures are complicated by CSF leakage, but most of them are managed conservatively [8]. A breach of the arachnoid, dura, and bone will determine a route of egress of the CSF through the mastoid cells and further on to oto-rhinorrhea or rhinorrhea if the tympanic membrane is intact [8]. We presume that maneuvers that change the pressure of the CSF, like Valsalva, were probably responsible for an intermittent shunt between the posterior fossa and the mastoid cells in our patient.

The first otorhinolaryngology examination showed evidence of haemotympanum, which was interpreted in the context of epistaxis. The bleeding gradually abated, and clear liquorrhea could be seen at a follow-up examination. Epistaxis has been previously reported in patients with headaches, and the presumed mechanism might be an aberrant activation of the trigeminovascular system or a transient increase of venous pressure, which could also explain bleeding from superficial bridging veins with subsequent subarachnoid hemorrhage [9].

Remission of the patient’s symptoms with conservative therapy was concordant with the abating of oto-liquorrhea on follow-up ENT examination and is in general line with his clinical history of infrequent spells of orthostatic headache and dizziness. We considered this clinical course suggestive of the intermittent nature of the dural leakage. Dynamic optic nerve sheath diameter measurements by ultrasound can be used to prove the opening or closing of dural fistulas responsible for CSF leaks, but it could not be performed at that moment in our hospital [10].

Although we did not perform dynamic myelography to rule out a spinal CSF leak, the brain MRI changes together with the findings of the otorhinolaryngology examination strongly point towards a cranial CSF leak leading to intracranial hypotension syndrome in our patient.

## Conclusion

We report the case of a patient with intermittent cranial cerebrospinal fluid leak and intracranial hypotension syndrome. The traumatic nature of his old temporal bone fracture makes the interpretation of this case as “spontaneous” intracranial hypotension disputable. However, the onset of symptoms years after the head trauma and their paroxysmal nature would have classified this syndrome as “spontaneous” if no cranial dural leak would have been found. Therefore, we believe that cranial cerebrospinal fluid leaks should be searched for in selected patients with spontaneous intracranial hypotension syndrome.

### Acknowledgments

#### Ethical approval

This case report was written in accordance with the legal and ethical requirements of the University Emergency Hospital Bucharest, Romania.

#### Consent to participate

Written informed consent was obtained from the patient.

#### Data availability

Further data are available from the corresponding author on reasonable request.

#### Conflict of interest

The authors declare that there is no conflict of interest.
